# A new mechanism for spatial pattern formation via lateral and protrusion-mediated lateral signalling

**DOI:** 10.1098/rsif.2016.0484

**Published:** 2016-11

**Authors:** Zena Hadjivasiliou, Ginger L. Hunter, Buzz Baum

**Affiliations:** 1 Centre for Mathematics, Physics, and Engineering in the Life Sciences and Experimental Biology, University College London, London WC1E 6BT, UK; 2 Department of Genetics, Evolution and Environment, University College London, London WC1E 6BT, UK; 3 MRC-Laboratory for Molecular Cell Biology, University College London, London WC1E 6BT, UK; 4 Institute of Physics of Living Systems, University College London, London WC1E 6BT, UK

**Keywords:** patterning, protrusion signalling, Notch, Delta, lateral inhibition

## Abstract

Tissue organization and patterning are critical during development when genetically identical cells take on different fates. Lateral signalling plays an important role in this process by helping to generate self-organized spatial patterns in an otherwise uniform collection of cells. Recent data suggest that lateral signalling can be mediated both by junctional contacts between neighbouring cells and via cellular protrusions that allow non-neighbouring cells to interact with one another at a distance. However, it remains unclear precisely how signalling mediated by these distinct types of cell–cell contact can physically contribute to the generation of complex patterns without the assistance of diffusible morphogens or pre-patterns. To explore this question, in this work we develop a model of lateral signalling based on a single receptor/ligand pair as exemplified by Notch and Delta. We show that allowing the signalling kinetics to differ at junctional versus protrusion-mediated contacts, an assumption inspired by recent data which show that the cleavage of Notch in several systems requires both Delta binding and the application of mechanical force, permits individual cells to act to promote both lateral activation and lateral inhibition. Strikingly, under this model, in which Delta can sequester Notch, a variety of patterns resembling those typical of reaction–diffusion systems is observed, together with more unusual patterns that arise when we consider changes in signalling kinetics, and in the length and distribution of protrusions. Importantly, these patterns are self-organizing—so that local interactions drive tissue-scale patterning. Together, these data show that protrusions can, in principle, generate different types of patterns in addition to contributing to long-range signalling and to pattern refinement.

## 1. Introduction

Patterning is key to the development of complex multicellular organisms. Indeed, the organization of initially uniform cells into regular motifs such as stripes or spots has been widely documented across species and scales. Some examples include the salt-and-pepper patterns of bristle precursor cells in the *Drosophila* fly [1], the pigmentation stripes of zebrafish [2] and branching during organ development [3]. These beautifully organized patterns emerge through the spatial differentiation of genetically identical cells that take up distinct developmental fates according to their position in the developing organism [4]. How initial symmetry is broken to give rise to these patterns remains an open question.

Several theories have been proposed to explain cellular pattern formation, most notably Turing's reaction–diffusion model [5]. Turing showed that a slowly diffusing activator and a fast diffusing inhibitor can generate a range of periodic patterns whose properties (e.g. density and regularity) will depend on the decay length of the activator and inhibitor molecules themselves, the so-called ‘morphogens’. Although classical reaction–diffusion systems can generate diverse patterns, it is becoming increasingly evident that signalling interactions mediated via direct cell–cell contact (juxtacrine signalling) can also lead to self-organized tissue patterning [6–8]. Importantly, juxtacrine interactions are not restricted to immediate neighbours: cells can exchange signals at a distance from one another through long cellular protrusions [9]. This has long been known for neurons, but mounting evidence suggests that signalling through cellular protrusions is a more general feature of animal cells [6].

**Figure 1.**
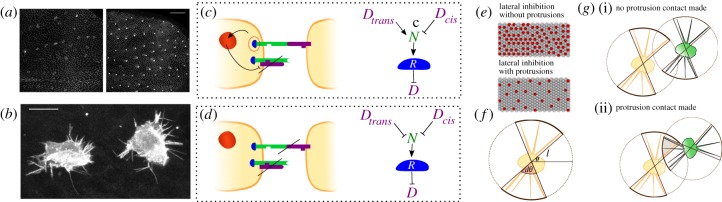
(*a*) Patterning of the *Drosophila* notum. *Drosophila* pupal hemi-notum expressing shotgun-GFP (shgGFP, labels all apical cell boundaries) and neuralized-GMCA (GFP-tagged F-actin reporter expressed in SOPs). At the onset of patterning (12 h after pupariation (AP)), few cells are neuralized-GMCA positive. By 24 h AP, the tissue is patterned with a sparse, ordered distribution of SOPs. Scale bar, 50 µm. Anterior is to the right in all images. (*b*) Visualization of basal protrusions in SOPs. Basal *z*-projections (less than or equal to 10 µm) of SOPs expressing a GFP-tagged F-actin reporter under the *neuralized*-GAL4 driver. Scale bar, 10 µm. (*c*) Schematic of Delta (purple) Notch (green) and Notch reporter (*R*) interactions. When Delta (*D*) binds to Notch (*N*) in *trans*, it activates the intracellular part of the Notch receptor (*R*) to inhibit Delta production in the receiving cell. Notch–Delta interactions within the same cell (*cis* interactions) lead to inactivation of both the ligand and the receptor. (*d*) Notch–Delta interactions engaged in *trans*-inhibition instead of *trans*-activation of the Notch receptor. (*e*) Notch–Delta-mediated lateral inhibition gives rise to salt-and-pepper spatial patterns. Protrusion signalling results in sparser patterns. (*f*) Schematic of protrusions in our model. (*g*) Modelling protrusion polarization and interactions. (i) A scenario where the protrusions of two cells are within range of one another, but do not overlap due to polarization. (ii) Successful protrusion-mediated interactions between two cells. The area of potential contact is shaded. More details are provided in the Methods section.

Protrusion-mediated signalling is implicated in patterning in several developmental systems. For example, the stripy pigment patterns in zebrafish are formed through planar contact-mediated interactions between three different types of motile cells. Here, short junctional contacts and long-range protrusion-mediated contacts between different cell types lead to the mutual attraction or repulsion among cells that self-organize into stripes [2,10]. The regularly spaced bristle precursor cells in the *Drosophila* notum also rely on the combination of direct and protrusion-mediated signals [1,11]. In this system, cells expressing high levels of the Delta ligand inhibit the expression of Delta, via Notch activation, in other cells within their reach. The result is a regularly spaced pattern of individual cells expressing high levels of Delta in a sea of cells expressing low levels of Delta (figure 1*a,b*) [1,11]. In this way, contact-mediated signalling can lead to the generation of periodic patterns in an initially homogeneous tissue without the requirement for cell motility or a pre-pattern.

Although spot patterns of varying density and regularity can be obtained by modulating the protrusion dynamics and length [1], it is not clear whether, in the absence of molecular diffusion, more complex patterns can emerge. To test this idea, here we develop a model of lateral inhibition with feedback based on Notch–Delta signalling to explore the capacity of contact-mediated signalling to generate diverse patterns. For this analysis, we assume that cellular interactions occur both over a short range, where they are mediated by cell–cell junctional contacts, and at long range, via protrusions. In addition, building on a previous theoretical framework of lateral inhibition [1,12], we allow the kinetics of signalling at short range junctional contacts and signalling via protrusions contacts to differ. As our results show, this has surprising consequences. This type of juxtacrine signalling leads to a variety of self-organizing patterns, ranging from sparsely or densely spaced stripes, labyrinths and radii, to clusters and regular salt-and-pepper patterns of different density. This we suggest represents a new mechanism of pattern formation. Finally, we explore the role of the signalling dynamics, protrusion length and directionality in the patterning process and consider its broader implications for our understanding of developmental patterning.

## 2. Model outline

The patterning of cell fates across a tissue is a key aspect of animal development [13]. One of the best understood examples of patterned cell fate determination is lateral inhibition mediated by the Notch–Delta signalling pathway [1,2,12,14]. This occurs when Delta ligands on the surface of one cell bind to Notch receptors expressed by its direct neighbours. Under certain conditions, this can trigger Notch cleavage, enabling the Notch intracellular domain to enter the nucleus to trigger downstream changes in gene expression. Importantly, this includes the inhibition of Delta expression, a feature of the system that can drive symmetry breaking (figure 1*c*). In this work, we explore the capacity of contact-mediated lateral inhibition systems to generate diverse patterns. Although we use Notch–Delta signalling as a basis for this model, our conclusions will apply to other systems that use a similar logic.

**Figure 2.**
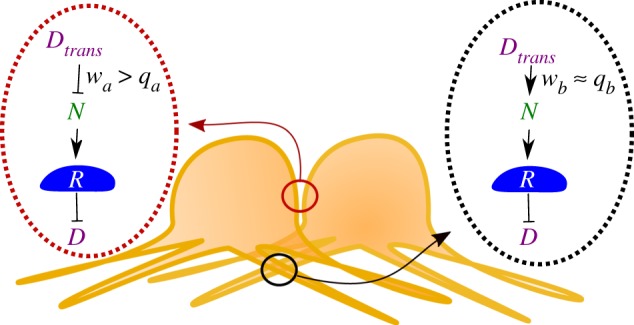
Schematic of possible signalling interactions between cells in the model. Here, Notch–Delta signalling at the junctional contacts does not always lead to activation of the Notch receptor (*R*). Instead, the receptor (*N*) can bind to Delta (*D*) in *trans* without becoming activated (*w_a_* > *q_a_*). Interactions mediated through the protrusions are more likely to lead to receptor activation (*w_b_* ≈ *q_b_*).

We model a tissue comprising *M* × *M* cells, packed in a hexagonal lattice. Signalling interactions can occur only between cells that are in physical contact, either directly at junctional contacts or through cellular protrusions extended away from a cell's centre. We assume that the tissue is initially homogeneous with all cells expressing low levels of both the Delta ligand (*D*) and its receptor Notch (*N*), which we initiate using Gaussian noise (see Methods). The expression levels of the intracellular Notch reporter (*R*) are initially set to 0. We model the expression dynamics of Delta and Notch by building on a previously published mathematical framework [12,15]:2.1
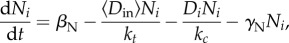
2.2
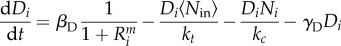
2.3
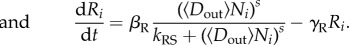
Although several models of Notch–Delta signalling and lateral inhibition have been developed [1,12,14,16], we choose this particular formulation because it allows us to track the free Notch receptors on the cell membrane as well as the amount of active Notch signal within each cell. This ability to disentangle Notch receptor binding from the induction of Notch signalling is central to our model analysis and findings. The parameters *β*_N_*, β*_D_*, β*_R_ and *γ*_N_*, γ*_D_*, γ*_R_ are the production and degradation rates of Notch, Delta and the intracellular Reporter of Notch signalling, respectively. The constants *k_t_* and *k_c_* determine the strength of Delta–Notch binding in *trans* (between cells) and in *cis* (within a single cell), respectively, and *k*_RS_ is the dissociation constant of the intracellular signal. The total amount of incoming Delta and Notch is given by2.4
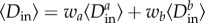
and2.5
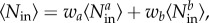
where *w_a_* and *w_b_* are used to implement differential weighting for the incoming signal at junctional contacts and protrusion-mediated contacts. 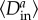 is equal to the total incoming Delta summed over all cells that make ***junctional*** contacts with the cell of interest. Similarly, 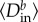 is the total incoming Delta summed over all cells that contact the cell of interest via ***protrusions***. Note that there is a third type of contact, in which the protrusion of one cell contacts the cell body of another. In these cases, we assume that the signalling contact made is equivalent to a protrusion-mediated contact, if Delta is present on protrusions, and that this can sample the full set of Notch receptors on the receiving cell. Otherwise, if Delta is present on the cell body but not protrusions, the contact made is junctional. The distinction in signal strength captured by differences in *w_a_* and *w_b_* is used to represent both different concentrations of the signal at the cell membrane and protrusions [1,17], and/or differences in the efficiency of Delta–Notch binding at these different types of contact. As an additional assumption, not all bound Delta–Notch molecules lead to an intracellular signal under the model. Instead, Delta can bind to Notch in another cell without leading to activation (figure 1*d*). This amounts to inhibition in *trans* (figures 1*c* and 2)—a process that has not to our knowledge been previously considered in this context—but which nicely captures the requirement for mechanical force for Notch cleavage and signalling in many experimental systems [18,19]. We further discuss the potential role of mechanical force in our model at the end of this article. Here, we do not directly model mechanical force, but we simply allow the efficiency of Notch signalling to vary by defining the proportion of incoming Delta leading to activation of the Notch receptor in the receiving cell (figures 1*d* and 2),2.6
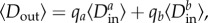
where 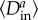 and 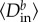 are as defined above and 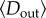 is equal to the total amount of bound Delta that leads to activation of the intracellular part of the Notch receptor. It follows from this formulation that the proportion of Delta molecules bound in *trans* that leads to a Notch signal in the receiving cell is equal to *q_a_*/*w_a_* for a junctional contact, *q_b_*/*w_b_* for protrusion-based interactions. Thus, *q_a_* and *q_b_* are bounded by *w_a_* and *w_b_*, respectively.

We model protrusions by defining the protrusion length for each cell, *l*, and the directionality of the protrusions by defining an angle of polarization coupled to an arc opening (*θ*, *dθ*) as shown in figure 1*f*. To model cells exerting protrusions in all directions, we simply let *θ* take any value and set *dθ* = *π*. Protrusion signalling is allowed for cells whose protrusions are within reach of one another (see Methods for details).

When we set *w_a_* = *q_a_* = 1, *w_b_* = *q_b_* = 0.3 and *l* = 2.3 × (cell diameter)—close to the measured parameters for Notch–Delta-mediated lateral inhibition in the fly notum [1,17]—we obtain a pattern resembling the wild-type *in vivo* bristle spacing (figure 1*a*; [1]). Naturally, this pattern is sparser than ones obtained without protrusions, because protrusions increase the range of signalling (figure 1*e*; [1,16]).

## 3. Differential signalling efficiency at short-range and protrusion-mediated contacts expands pattern space

**Figure 3.**
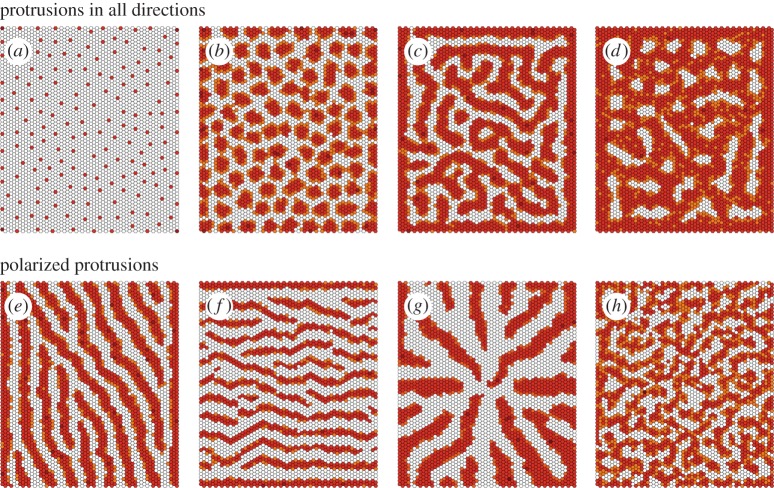
Possible patterns using our model and setting *w_a_* > *q_a_* and *w_b_* ≈ *q_b_*, without a bias in the protrusions directionality (*a*–*d*) and with polarized protrusions (*e*–*h*). The star pattern in (*g*) is obtained by allowing cells to exert protrusions perpendicular to radii focused in the tissue centre. The labyrinthine pattern in (*h*) is obtained by assuming that cells exert protrusions along a random but not necessarily fixed direction. The parameter values for each plot are given in table 1. Baseline parameters common across simulations are detailed in the Methods section. Colour scheme: white, *D* < 0.005; orange, 0.005 < *D* < 0.05; light brown, 0.05 < *D* < 0.5; dark brown, *D* > 0.5. The values of Delta (*D*) used for the colour scheme were normalized by dividing across by the maximum Delta value for any given expression matrix.

Although the spacing, density and regularity of the salt-and-pepper patterns such as those seen in the *Drosophila* notum can be modulated by changing protrusion length and/or dynamics (figures 1*e* and 3*a*; [1]), it is not clear how more diverse patterns (e.g. pigment stripes in zebrafish, *Drosophila* wing veins) can be obtained through lateral signalling alone without the inclusion of complex interactions between cells of several different types [2,7]. Nevertheless, by increasing the relative amount of Delta signalling at direct, compared with protrusion-mediated contacts (*w_a_* > *w_b_*), while decreasing the binding-to-activation efficiency for direct contacts (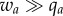; figure 2), we were able to obtain patterns more complex than regularly spaced dots (figure 3). These range from regularly spaced clusters of cells to labyrinthine patterns (figure 3*a–d*). This shows that it is possible to induce a much broader set of patterns than expected using lateral inhibition-type signalling.

**Table 1.** Parameter values varied across simulations. Baseline parameters that were common in all simulations are provided in the Methods section.

figure	*w_a_*	*q_a_*	*w_b_*	*q_b_*	*l*	*θ*	*dθ*
figure 3*a*	1	1	1	1	7	—	*π*
figure 3*b*	1	0.01	0.1	0.025	7	—	*π*
figure 3*c*	1	0.01	0.01	0.01	7	—	*π*
figure 3*d*	1	0.01	0.01	0.005	7	—	*π*
figure 3*e*	1	0.01	0.1	0.05	7	*π*/6	*π*/20
figure 3*f*	1	0.01	0.2	0.2	7	*π*/2	*π*.20
figure 3*g*	1	0.01	0.1	0.05	12	—	*π*/20
figure 3*h*	1	0.01	0.1	0.05	7	—	*π*/20
figure 4*a*	1	0.01	0.1	0.05	—	*π*	*π*/20
	1	0.01	0.1	0.1	—	—	*π*
figure 4*b*	1	0.01	0.1	0.05	10	*π*	—
	1	0.01	0.1	0.1	10	—	—
figure 6	1	—	—	1	7	*π*/6	*π*/20
figure 7*a*	0.5	0.25	0.5	0.025	9	*π*/6	*π*
figure 7*b*	1	0.3	0.25	0.025	7	*π*/6	*π*
figure 7*c*	1	0.25	0.25	0.025	7	*π*/6	*π*
figure 7*d*	0.5	0.5	0.5	0.05	11	*π*/6	*π*/40
figure 7*e*	0.5	0.5	1	0.1	7	*π*/6	*π*/20
figure 7*f*	1	1	2	0.2	11	*π*/6	*π*/100

As the model is based upon lateral inhibition, how do clusters of cells high in Delta emerge under these conditions? This behaviour arises from the assumption that, at short-range contacts, a large complement of the set of Notch receptors expressed by a cell neighbouring a Delta-expressing cell are likely to be occupied by Delta without Notch being cleaved. As a consequence of this low-efficiency signalling (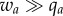; figure 2), which we call *trans* inhibition, these ‘first cell neighbours’ are rendered non-responsive to incoming Delta signals, enabling them to express Delta ligand (figure 1*d*). As, under the first set of test assumptions used, Delta is assumed to have a high binding-to-signalling efficiency when acting on protrusions (*w_b_* ≈ *q_a_*; figure 2), these cells with sequestered Notch are then able to signal to more distant secondary and tertiary neighbours that express the Notch receptor (figure 2; equations (2.1)–(2.5)). This leads to a complex interplay between signalling at junctional cell–cell contacts (which can sequester Notch receptors without inducing Notch cleavage to generate regions of tissue that are crowded with adjacent Delta-expressing cells) and protrusion-mediated signalling (which may induce the binding and cleavage of Notch receptors to inhibit Delta production over a longer range), which we investigate in more detail in a latter section. This behaviour is strikingly different from that induced by the process of *cis*-inhibition, whereby Delta and Notch within the same cell bind one another in an inhibitory manner. In our model, as in published models [12,14,15], *cis*-inhibition functions primarily to sharpen and speed up the process of lateral inhibition-mediated tissue patterning.

The way our model is formulated also allows protrusions to be polarized in a given direction in space (figure 1*f*). As animal cells frequently generate directional protrusions in response to local cues, this would appear to be an assumption worthy of testing [20]. This protrusion polarization further expands the pattern space—leading to the formation of stripes of differentiated cells perpendicular to the direction of protrusion polarization (figure 3*e,f*). In addition, radii are generated when protrusions are polarized perpendicular to the lattice centre (figure 3*g*). When the polarity cue governing protrusion directionality is made cell autonomous, so that the direction is allowed to vary between cells (as is often seen to be the case for structures in PCP (planar cell polarity) mutants [21]), we obtain less organized patterns resembling labyrinths (figure 3*h*). Taken together, these results show that a wide variety of patterns from dots and clusters to stripes, labyrinths and radii can all be generated simply by varying the efficiency of signalling interactions at junctional and protrusion-mediated contacts and protrusion directionality.

**Figure 4.**
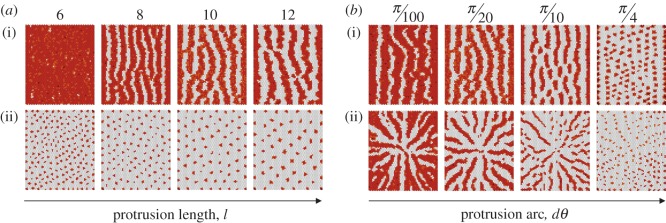
Exploring the impact of (*a*) the protrusion length, *l*, and (*b*) the strength of the protrusion polarization bias, *dθ*, on the overall pattern. The value of *l* and *dθ* is indicated on the top of each panel. Protrusions are polarized with *θ* = *π* in *a*(i) and *b*(i) are projected in all directions in *a*(ii) and are projected according to a radial symmetry in *b*(ii). Further parameter values for each plot are given in table 1. Baseline parameters common across simulations are detailed in the Methods section. Colour scheme: white, *D* < 0.005; orange, 0.005 < *D* < 0.05; light brown, 0.05 < *D* < 0.5; dark brown, *D* > 0.5. The values of Delta (*D*) used for the colour scheme were normalized by dividing across by the maximum Delta value for any given expression matrix.

As protrusion signalling is key to patterning in our model, we next tested the impact of changing protrusion length and polarization in the system. When we varied the average protrusion length, *l*, we noted that the pattern density changed (figure 4*a*). Long protrusions can span larger distances and so lead to sparser patterns, whereas shorter protrusions result in more dense patterns (figure 4*a*). Hence, the pattern wavelength depends on the protrusion range.

Protrusion directionality is also characterized by the arc opening, *dθ* (figure 1*f*). A larger *dθ* means that protrusions are less well polarized. So, for example, setting *dθ* = *π* is equivalent to setting up a system in which the distribution of protrusions is randomized, whereas by setting *dθ* = 0 protrusions can be made to project along a fixed line that is defined by *θ* (figure 1*f*). Using our model to generate patterns for different values of *dθ*, we found that the pattern density and regularity change with this parameter (figure 4*b*). A smaller *dθ* results in better-aligned and more pronounced stripes, labyrinths and radii, whereas the regularity of the pattern is reduced for larger *dθ* (figure 4*b*). Consequently, variations in the pattern density and regularity can be achieved by modulating the protrusion length and directionality.

**Figure 5.**
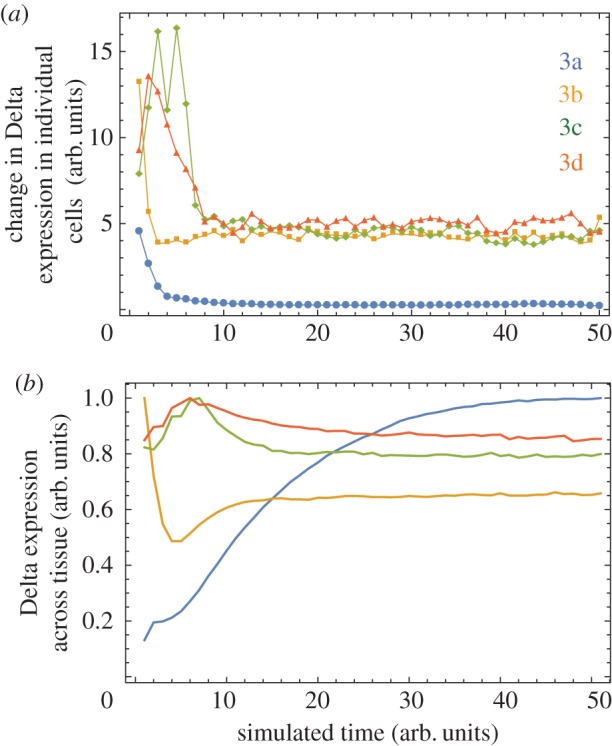
Quantifying pattern stability across simulations used for figure 3*a*–*d*. (*a*) The change in the Delta expression of *individual* cells between time intervals of 1 arb. units in the simulation. The change is calculated as the Euclidian distance between the normalized *N* × *N* matrices holding the Delta expression for all cells in the hexagonal lattice, from time *T* to time *T* + 1. In this way, panel (*a*) is a quantification of the change in Delta expression in *individual* cells over time. (*b*) Mean Delta expression across the tissue over time. This is computed by averaging the Delta expression across cells in the hexagonal lattice (tissue) at each time step. In this way, panel (*b*) provides a quantification of the overall pattern density over time. Each line shown is averaged over 10 independent simulations.

During development, although patterns take time to emerge, they ultimately reach a stable state as cells become committed to a fixed fate. This led us to quantify pattern stability by measuring the *change* in the overall expression of Delta in individual cells over time. This is shown in figure 5*a* for the patterns in figure 3*a–d*. The pattern in figure 3*a* where *w_a_* = *q_a_* and *w_b_* = *q_b_* quickly stabilizes and the expression in individual cells barely changes over time. This is not true for the patterns depicted in figure 3*b–d*, where cell states changes are still observed at late times (simulations were run up to time *T* = 55(arb. units)). Although, the system may not reach stasis in these cases, the apparent instability observed (figure 5*a*) is due to flickering in the expression levels of Notch and Delta in a subset of cells (electronic supplementary material, figure S1) as they flip-flop from one cell fate into the other—a process that helps to ensure that the overall pattern density and the qualitative pattern stabilize at later times (beyond *T* = 40). Thus, in these cases, the overall expression (averaged over the entire tissue) reaches a maximum and stable value (figure 5*b*).

## 4. The role of binding-to-activation rates

**Figure 6.**
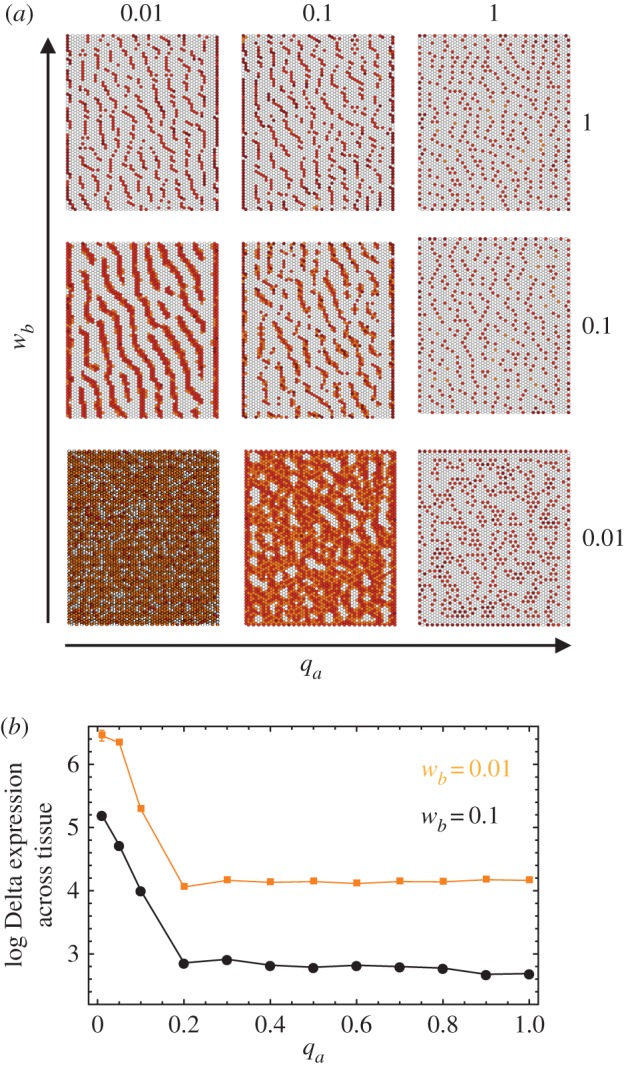
Varying *w_b_* and *q_a_*. (*a*) The resulting pattern for different (*w_b_*, *q_a_*) pairs and assuming that *w_a_* = 1 and *q_b_* = *w_b_*. (*b*) The log of the tissue average Delta expression against *q_a_* for *w_b_* = 0.1 and *w_b_* = 0.01. The pattern becomes more dense for smaller *q_a_* and *w_b_*, in agreement with the qualitative picture in (*a*). Additional parameter values and baseline parameters common across simulations are detailed in table 1 and in the Methods section. Colour scheme: white, *D* < 0.005; orange, 0.005 < *D* < 0.05; light brown, 0.05 < *D* < 0.5; dark brown: *D* > 0.5. The values of Delta (*D*) used for the colour scheme were normalized by dividing across by the maximum Delta value for any given expression matrix.

The relative efficiency of signalling at junctional contacts and protrusion-mediated contacts is critical for patterning in this model. On the one hand, the ability of Delta ligands at a junctional contact (low-efficiency signalling) to sequester inactive Notch receptors on the surface of its neighbours (determined by *w_a_*/*q_a_*) regulates Delta expression; the larger the ratio *w_a_*/*q_a_*, the faster the generation of large patches of Delta-expressing cells. On the other hand, the size of these patches will be limited by the action of high-efficiency protrusion-mediated Delta signalling. The amount of incoming Delta signal received by a protrusion expressing Notch, defined by *w_b_*, and the efficiency of protrusion signalling, defined by *w_b_*/*q_b_*, therefore, determine the rate at which the Notch signal inhibits Delta production. So far we assumed *w_b_* ≈ *q_b_* (figure 2). Hence, *w_b_* defines the strength of Notch receptor activation and the inhibition of Delta production, and the relative values of *w_a_*/*q_a_* and *w_a_*/*w_b_* determine whether it is possible to establish a pattern at all. This is illustrated in figure 6*a*, where *w_a_* is set equal to 1 and *q_a_* and *w_b_* are varied: the pattern becomes increasingly dense as *w_b_* and *q_a_* decrease. When *w_a_* = *q_a_*, i.e. when signalling at junctional contacts always leads to receptor activation (figure 1*c*), we no longer see solid stripes. Instead, a dotted pattern emerges, where the density of the dots is maximized in the direction perpendicular to the protrusion polarization. This was confirmed by averaging the results of more than 10 independent runs (figure 6*b*).

**Figure 7.**
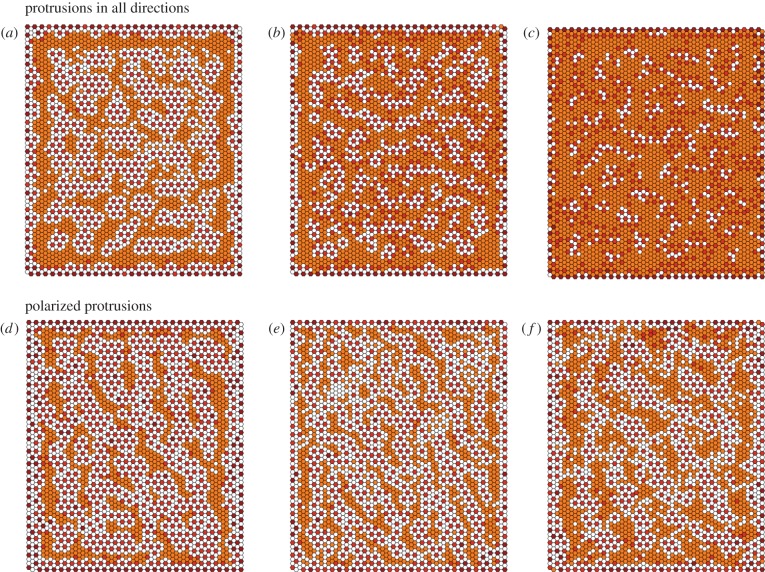
Possible patterns assuming that *w_b_* > *q_b_* and *w_a_* ≈ *q_a_*, without a bias in the protrusions directionality (*a*–*c*) and with polarized protrusions (*d*–*f*). The parameter values for each plot are given in table 1. Baseline parameters common across simulations are detailed in the Methods section. Colour scheme: white, *D* < 0.005; orange, 0.005 < *D* < 0.05; light brown, 0.05 < *D* < 0.5; dark brown, *D* > 0.5. The values of Delta (*D*) used for the colour scheme were normalized by dividing across by the maximum Delta value for any given expression matrix.

We have thus far assumed *w_a_* > *q_a_* and *w_b_* ≈ *q_b_*, that is Notch–Delta interactions at junctional contacts are more likely to lead to receptor activation than protrusion-mediated interactions (figure 2). We now ask whether reversing this assumption has an impact on pattern formation. Letting *w_a_* ≈ *q_a_* and *w_b_* ≫ *q_b_* we were able to generate patterns that are qualitatively distinct to those seen so far (figure 7). In particular, an underlying dotted pattern develops, similar to that seen in the absence of protrusion-mediated signalling. Overlaid on this are cells of intermediate Delta expression that pattern into noisy labyrinths, clusters or stripes according to the protrusion directionality (figure 7). How are these patterns generated and why does reversing the relationship of (*w_a_*, *q_a_*) and (*w_b_*, *q_b_*) lead to these changes? Setting *w_a_* = *q_a_* means that cells expressing high levels of Delta quickly inhibit Delta production in their primary neighbours, generating the baseline dotted pattern. In addition, protrusion-mediated signalling is inhibited by the sequestration of inactive Notch receptors within their reach (since *w_b_* > *q_b_*). As a consequence, cells within reach of the protrusions of several Delta cells become refractory to lateral inhibition—leading to areas of intermediate Delta expression. In this way, our model produces patterns beyond those that are typical of classical reaction–diffusion systems.

## 5. Discussion

How can initially uniform tissues self-organize into complex spatial patterns without a chemical pre-pattern or the ability of cells to migrate? In this work, we propose that a lateral inhibition mechanism comprised a single ligand–receptor pair based on Notch–Delta signalling can generate a wide set of patterns. These vary from regularly spaced dots and clusters to stripes, radii, labyrinths and more—extending the set previous described for typical Notch–Delta models of lateral inhibition. Furthermore, our model shows that complex tissue-wide patterns can be generated by direct cell–cell contacts alone. Importantly, although this work is based on Notch–Delta signalling, our approach is general and is therefore applicable to other systems that combine contact-mediated signalling with feedback.

Key to our model is the assumption that the kinetics of Notch–Delta signalling differ at junctional contacts at the cell interface and protrusion-mediated contacts. In particular, Delta can bind to Notch at junctional contacts without this being translated into a high probability of receptor activation. This assumption is central because it allows patches of neighbouring cells to express high levels of Delta. This process, which we term *trans* inhibition, has not to our knowledge between described previously. At the same time, protrusion-mediated signalling in the model activates the Notch receptor with a high probability to enforce lateral inhibition. The balance between these two processes is equivalent to a balance between local self-enhancement and long-range inhibition, conditions shown to be necessary and sufficient for periodic pattern formation [22,23]. Interestingly, more unusual types of pattern are generated when these assumptions are reversed, so that junctional contacts between cells activate lateral inhibition signalling, whereas protrusion-mediated contacts sequester the Notch receptor. Thus, complex patterns could be generated in tissues via contact-mediated signalling, if (i) the amount of Delta differs at junctional contacts versus protrusions [1] and (ii) the efficacy of signalling can be modified [24,25]. Importantly, this may help to explain how it is that Notch–Delta signalling functions to establish patterned lines as well as spots in some developing tissues, e.g. the fly wing [26].

A question that naturally follows is how can a single cell enforce signalling in a distinctive manner at different locations. Endocytosis is likely to play a role, as it generates a force necessary for Delta-dependent Notch receptor activation [19,27,28]. Without this, the Notch receptor may bind to Delta without becoming activated [27]. Similarly, endocytosis has been proposed to activate or inactivate many other receptor-based signals [29]. Whether or not endocytosis is also key for signalling at the protrusions is debatable. Instead, other factors could be more important here, such as the force generated when actin-based protrusions extend and retract. In this way, the efficacy of signalling could be locally modified by polarization of the contractile machinery, a common feature of most animal cells. Further *in vivo* studies should elucidate whether such mechanisms exist and, if so, whether they function to aid biological patterning.

Although diverse patterns can be stable under Notch–Delta signalling with strong *cis-*inhibition [14], the *de novo* generation of patterns more complex than regularly spaced dots through Notch–Delta signalling alone is, to our knowledge, novel to this work. A recent study showed that the ability of signalling cells to control the spatial distribution of the ligand along their protrusions could give rise to more complex patterns such as stripes and clusters of cells [30]. This is an interesting possibility, although it can be constrained by the requirement for precise control of the ligand distribution along individual protrusions. Our model suggests that the effectiveness of protrusion signalling can be variable as one moves away from the signalling cell due to signalling dynamics at different types of cell–cell contact. Other models of lateral inhibition, for example those based on spatial and temporal noise inhibitory thresholds, can also give rise to more diverse patterns [31]. Interestingly, in these cases, the inhibitory thresholds function in a similar way to the model described here, because they enable cells to sum signals from different types of cell–cell contact. Similarly, some of the more unusual pattern motifs identified here (figure 7), resemble those seen in a recent study of probabilistic patterning through lateral inhibition [32]. In this way, this model, which is inspired by the observation that cells exhibit different types of Notch–Delta contacts, provides a plausible mechanism by which the conditions identified in more complex models of lateral inhibition/activation can be met and used in living organisms.

## 6. Methods

### 6.1. Computational methods

#### 6.1.1. Protein dynamics

We used a mathematical model to simulate lateral inhibition by Delta–Notch signalling. The model is defined by a set of coupled differential equations (equations (2.1)–(2.3)), which describe the dynamics of Notch (*N_i_*), Delta (*D_i_*) and a Reporter of Notch signalling (*R_i_*) for individual cells (*i* being the index for each cell).

The parameters *β*_N_, *β*_D_, *β*_R_ and *γ*_N_, *γ*_D_, *γ*_R_ are the production and degradation rates of Notch, Delta and the Reporter of Notch signalling, respectively. The constants *k_t_* and *k_c_* determine the strength of Delta–Notch interactions in *trans* and in *cis*, respectively, and *k*_RS_ is the dissociation constant for the intracellular signal. 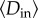 and 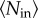 indicate the incoming Delta and Notch signal and are determined by summing the signal from all cells in contact with a given cell, scaled by a factor *w_a_* and *w_b_* for junctional and protrusion contacts, respectively (equations (2.4)–(2.5)). 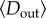 indicates the amount of Delta signal from neighbouring cells that contributes to Notch cleavage and thereby intracellular signal, and is determined by scaling incoming signal by a factor *q_a_* and *q_b_* for junctional and protrusion contacts respectively (equations (2.6)). A Gaussian noise term was applied to initiate protein consecrations (for *D* and *N*; *R* initially set to 0) and to the concentrations at each time step.

#### 6.1.2. Protrusion modelling

Basal protrusions were implemented as two-dimensional circular areas, extending from the centre of each cell (figure 1*f*). Radii were drawn from a normal distribution with mean = *l* and s.e. = 0.5. Protrusion polarization is implemented by assigning a value *θ* for the protrusion polarization coupled with an arc opening *dθ* as shown in figure 1*f*. This formulation does not explicitly consider individual protrusions but instead defines an arc opening within which protrusions are assumed to be projected. This is a valid approximation if several protrusions are exerted by individual cells. For any two cells (cell 1 and cell 2), spaced a distance apart *d* such that the sum of the length of their protrusions is below *d*, a signal occurs if the contact point is made within the arc determined by *θ* and *dθ* for both cells (figure 1*f*,*g*).

#### 6.1.3. Simulation parameters

The same baseline parameters used for all simulations: *β*_N_ = 100, *β*_D_ = 500, *β*_R_ = 300 000, *γ*_N_ = *γ*_D_ = *γ*_R_ = 1, *k_t_* = 2, *k_c_* = 0.5, *k*_RS_ = 10^7^, *m* = 2, *s* = 2. Initial expression values were sampled from a Normal distribution *N*(10^−3^*β*_N_, 10^−4^*β*_N_) and *N*(10^−3^*β*_D_, 10^−4^*β*_D_) for Notch and Delta, respectively. All *R*-values were initially set to 0. The cell radius *l* was set equal to 2 arb. units. The model was applied to a uniform hexagonally packed two-dimensional array of 60 × 60 cells. Simulations were performed by numerically solving equations ((2.1)–(2.3)) using the Euler method. Parameters that were varied between figures shown in table 1.

#### 6.1.4. Colour scheme in patterning figures

To use the same colour scheme across figures, we normalized the Delta expression matrix for each individual simulation so that no value exceeds 1. We did this by dividing by the maximum Delta expression within the expression matrix of each individual pattern. We then used the following colour scheme: white, *D* < 0.005; orange, 0.005 < *D* < 0.05; light brown, 0.05 < *D* < 0.5; dark brown, *D* > 0.5.

### 6.2. Experimental methods

#### 6.2.1. Fly strains

Fly stocks were maintained at 18°C. Shotgun-GFP, neuralized-GMCA (GFP-actin binding domain of moesin); neuralized-GAL4; UAS-GMCA. Flies obtained from Bloomington Drosophila Stock Centre (Bloomington, IN, USA) and the Baum Laboratory.

#### 6.2.2. Imaging

White pre-pupae (0 h AP) were picked and aged at 25°C (for 12 h) or 18°C (for 24 h), then dissected for imaging at 12 h AP, as previously published [33]. Live pupae were imaged on a Leica SPE or SP8 confocal microscope. Imaging datasets were analysed and processed using FIJI/ImageJ (NIH).

## Supplementary Material

Fig. S1
