# Supplementary figures and images for: A new mechanism for spatial pattern formation via lateral and protrusion-mediated lateral signalling
Source: J R Soc Interface. 2016 Nov;13(124):20160484. doi: 10.1098/rsif.2016.0484 (PMC5134009; doi:10.1098/rsif.2016.0484)

$T = 1$  $T = 4$  $T = 5$  $T = 6$  $T = 7$  $T = 10$  $T = 15$  $T = 20$  $T = 30$  $T = 40$  $T = 48$  $T = 49$  $T = 50$ 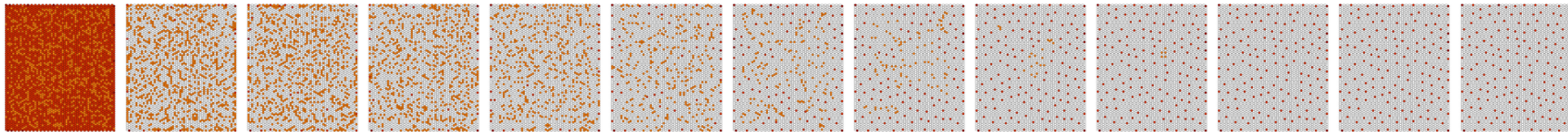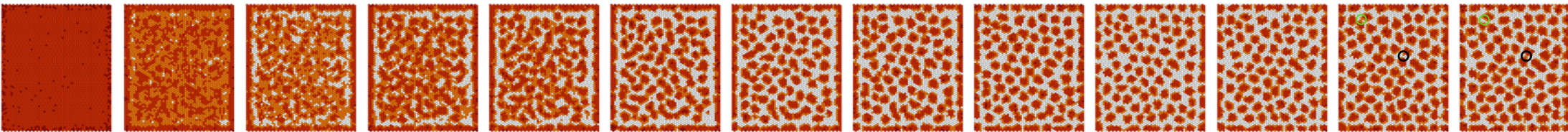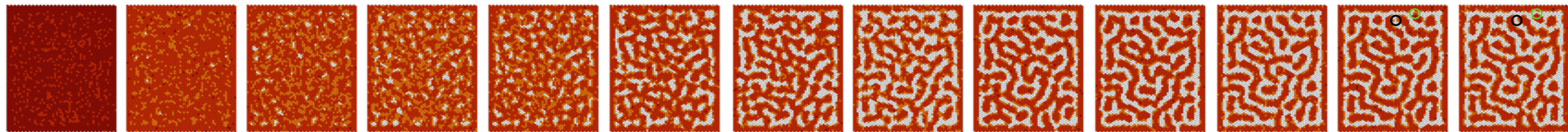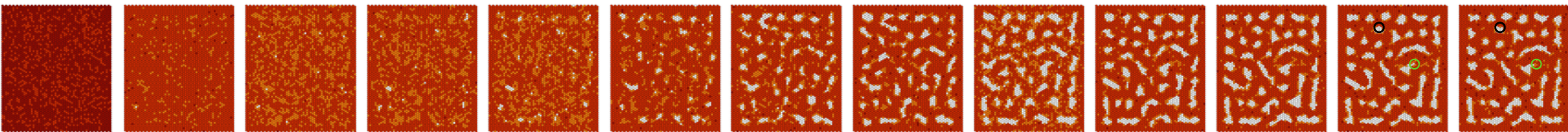

Supplement: Fig. S1 [file rsif20160484supp1.pdf]
